# A Comprehensive Narrative Review on the Evolving Role of Endoscopic Ultrasound in Focal Solid Liver Lesions Diagnosis and Management

**DOI:** 10.3390/diagnostics10090688

**Published:** 2020-09-11

**Authors:** Wisam Sbeit, Anas Kadah, Amir Mari, Mahmud Mahamid, Tawfik Khoury

**Affiliations:** 1 Department of Gastroenterology, Galilee Medical Center, Nahariya 22100, Israel; wisams@gmc.gov.il (W.S.); anask@gmc.gov.il (A.K.); 2 Faculty of Medicine in the Galilee, Bar-Ilan University, Safed 1311502, Israel; amir_mari@nazhosp.com; 3 Gastroenterology and Endoscopy Units, The Nazareth Hospital, EMMS, Nazareth 16100, Israel; 4 Department of Gastroenterology and Liver Diseases, Shaare Zedek Medical Center, Jerusalem 9103102, Israel; mmahamid@szmc.org.il; 5 Faculty of Medicine, Hebrew University of Jerusalem, Jerusalem 9112102, Israel

**Keywords:** endoscopic ultrasound (EUS), liver diseases, diagnosis, management

## Abstract

The implications of endoscopic ultrasound (EUS) have expanded considerably in recent years to cover more fields in invasive gastroenterology practice, as both an investigative and therapeutic modality. The utility of EUS in the diagnosis and management of focal liver lesions has gained a special attractiveness recently. The EUS probe proximity to the liver and its excellent spatial resolution enables real-time images coupled with several enhancement techniques, such as contrast-enhanced (CE) EUS. Aside from its notable capability to execute targeted biopsies and therapeutic interventions, EUS has developed into a hopeful therapeutic tool for the management of solid liver lesions. Herein, we provide a comprehensive state-of-the-art review on the efficacy and safety of EUS in the diagnosis and management of focal solid liver lesions. Medline/PubMed and Embase database searches were conducted by two separate authors (T.K. and W.S.), all relevant studies were assessed, and relevant data was extracted and fully reported. EUS-guided diagnosis of focal liver lesions by sonographic morphologic appearance and cytological and histopathological finding of biopsies obtained via fine needle aspiration/biopsy have been shown to significantly improve the diagnosis of solid liver lesions compared with traditional imaging tools. Similarly, EUS-guided treatment has been shown to consistently have excellent technical success, high efficacy, and minor adverse events. The evolving valuable evidences of EUS utility might satisfy the unmet need of optimizing management of focal solid liver lesions.

## 1. Introduction

Endoscopic ultrasound (EUS) was introduced as a diagnostic modality almost 40 years ago. Interestingly, it has rapidly gained popularity as an interventional and therapeutic tool in various gastrointestinal and liver disorders. The distinctive property of EUS to combine endoscopy and sonography in one hybrid device, together with the ability of bringing the transducer to a close proximity to the lesion of interest, facilitated the development of novel application of EUS. In recent years, new interventional advanced procedures have emerged, substituting surgical interventions, particularly in the elderly and amongst comorbid patients [1,2]. The reported high diagnostic yield, success, efficacy, and safety of interventional EUS procedures have paved their way towards the first line armamentarium, dealing with gastrointestinal diseases. Alongside the expanding use of radiological investigations, such as computerized tomography (CT) and magnetic resonance imaging (MRI), occasionally, practitioners find themselves facing incidental findings including a wide variety of liver lesion of uncertain behavior, mandating an effective and safe investigative tool. In recent years, an expanding volume of reports, describing EUS as a complementary tool in liver diseases diagnosis and treatment, have been published. In this review, we summarized the evolving evidence on the role of EUS in focal liver lesions diagnosis and management.

## 2. Unique EUS Properties Favoring Its Use in the Assessment of Focal Solid Liver Lesions

The EUS transducer proximity to the liver enables the endosonographer to perform a precise and accurate imaging study of the liver. Additionally, given the transducer proximity to the organ of interest, EUS can easily identify intervening structures and vessels, thus minimizing the rate of EUS-related adverse events, if fine needle aspiration (FNA) is planned [3,4]. Other advantages include the EUS ability to provide real-time elastography (RTE) and real-time doppler and contrast-enhanced (CE) images, which improve the diagnostic performance in the focal lesion [5] by providing semi-quantitative measurements of focal lesion stiffness and the vascular component of the lesion as recognized by color images [6,7].

## 3. Literature Search

Using the Medline/PubMed and Embase database, we performed a search with the keywords: EUS or endoscopic ultrasound or echoendoscopes and any of the following: liver or hepatic, solid, focal lesions, diagnosis, enhancement, fine needle aspiration or biopsy, treatment or therapeutic, ablation, ethanol injection, radiofrequency ablation (RFA), Neodymium: yttium-aluminum-garnet ablation, high-intensity focused ultrasound (HIFU), photodynamic therapy (PDT), and brachytherapy. The full articles were read, and data about technical success, diagnostic yield, efficacy, and safety were reported.

## 4. EUS-Guided Focal Solid Liver Lesions Diagnosis

With the widespread availability and use of imaging, especially CT and MRI, incidental asymptomatic focal liver lesions are not infrequently encountered, including benign and malignant liver lesion “incidentalomas” [8]. Frequently, to reach an exact diagnosis of focal liver lesions, additional specific imaging modalities, aspiration, biopsy, or combinations are needed. Elucidating the exact nature of these focal lesions is of tremendous significance as this may impact the management, stage, and prognosis [4]. In fact, the advantages of EUS discussed above enable practitioners to reach the precise diagnosis. The diagnostic role of EUS in focal liver lesions is mainly implemented in clinical practice to characterize incidental liver. This can be achieved by two diagnostic capabilities of EUS.

### 4.1. Visualization Capability

#### 4.1.1. B-Mode Traditional EUS

The role of EUS in the diagnosis of focal liver lesions has been mainly investigated in metastatic liver lesions and early hepatocellular carcinoma (HCC) [9,10,11]. The superiority of EUS over the other conventional imaging modalities (US, CT, and MRI) has been shown primarily in the detection of focal liver lesions of less than 1 cm [4,12,13], proposing EUS as an important imaging tool in suspected small liver metastasis in the setting of other primary malignancies. To date, few studies have been published in this field. Nguyen et al. was the first to publish a report regarding the ability of EUS to detect small liver metastasis missed by CT scans. Hence, EUS in this setting has an imperative role in detecting liver metastasis, not otherwise detected by other imaging modalities in patients suffering from other primary malignancies; an innovation that has a fabulous impact on patient management [14]. Sabbagh et al. assessed the performance of EUS in examining the left liver lobe among 24 patients with colorectal liver metastasis and showed that EUS was associated with higher detection of metastasis in liver segments II and III as compared to the standard imaging assessment [15]. Moreover, a similar study by Awad et al. showed the superiority of EUS over CT scans in uncovering occult small liver metastases that were not definitely diagnosed by CT scans [16]. Moreover, a study by Singh et al. has reported the diagnostic yield of EUS vs. CT in the detection of liver metastases among patients with newly confirmed diagnoses of pulmonary, pancreato-biliary, gastro-esophageal, and colonic malignancies, which revealed that the diagnostic accuracies of EUS and CT scans for hepatic lesions were 98% and 92%, respectively (*p* = 0.05). Additionally, EUS detected significantly higher numbers of metastatic lesions in the liver compared to the CT scans (40 vs.19 hepatic lesions, respectively; *p* = 0.008) [13]. In conclusion, the data currently available supports the use of EUS as a screening modality for liver metastasis, especially in the left liver lobe in the setting of primary malignancies.

#### 4.1.2. EUS-Related Enhancement Techniques

Aside from the better diagnostic yield of EUS in detecting small liver metastasis, the implementation of enhancement techniques in EUS examinations is used to better characterize focal liver lesions into either benign or malignant, thus precluding the need for performing a liver lesion-targeted biopsy. Enhancement techniques include elastography and ultrasound contrast agents (UCA), which have been initially used as a complementary tool in conventional ultrasound [17]. Recently, EUS elastography has been increasingly implemented as an important additive tool to optimize the diagnostic yield of EUS examination in the setting of focal liver lesions investigation due to its ability to differentiate between soft and solid mass components based on stiffness quantification [18]. This property constitutes a significant contribution to EUS as it represents a substantial additive tool to distinguish between malignant and benign focal liver lesions, as malignant lesions are much stiffer than benign lesions and surrounding parenchyma [19,20] A previous study has reported that the stiffness of malignant liver lesions is approximately 100 times higher than the surrounding normal tissue [19]. Similarly, a recent comparative study assessed patients with various gastrointestinal malignancies (HCC, cholangiocarcinoma, and liver metastases) as compared to benign gastrointestinal lesions. The authors showed a significantly stiffer level among the malignant lesions. A histogram cutoff value of 170 showed the best distinguishing ability of benign to malignant lesions with 92.5% sensitivity, 88.8% specificity, 88.6% accuracy, 86.7% positive predictive value, and 92.3% negative predictive value [20]. Moreover, the introduction of UCA either under Doppler by CE-EUS [21] or contrast harmonic-EUS (CH-EUS) has added significantly to the EUS-capability of detecting and characterizing focal liver lesions by improving the visibility of the microvascular architecture, thus allowing a differentiation between benign and malignant lesions [22,23,24] The unique property of UCA in focal liver lesions diagnosis takes advantage of the dual blood supply of the liver, which is divided into the arterial phase, lasting up to 30 s from injection during its enhancement and increasing progressively, the portal phase, lasting from 30–120 s, and the venous phase thereafter. Given that focal liver lesions have unique characteristics of vascular enhancement and washout, these techniques provide an excellent and promising tool to accurately define the lesion nature [25,26]. So far, few studies have been published in this field. Oh et al. reported the superiority of CH-EUS over traditional EUS in diagnosing liver metastasis in 30 patients and also showed that only 73.3% of patients were diagnosed with liver metastasis by traditional EUS, while this rate increased significantly to 93.3% among patients after performing CE-EUS with 100% technical success and no procedure-related complications [27]. Recently, Minaga et al. assessed the additive role of CH-EUS in the identification of metastasizing pancreatic adenocarcinoma to the liver over traditional EUS and a multidetector CT scan. They found that CH-EUS was associated with a significantly higher detection rate of left-lobe metastasis (diagnostic accuracy of 98.5% for CH-EUS compared to 91.1% for traditional EUS and 90.5% for CT scans). Moreover, CH-EUS-guided tissue acquisition led to excellent diagnostic accuracy, even in the case of a small lesion, less than 1 cm [28]. Finally, the data regarding EUS-related enhancement techniques mentioned above is still evolving; however, it is evident that these techniques bring a breakthrough promise, which can be clinically implemented into the daily clinical practice in suspected cases of focal liver lesions, thus leading to the optimization of patient management. Further large prospective studies are needed to definitely establish its role.

### 4.2. Tissue Diagnosis Capability

EUS-guided liver biopsy (EUS-LB) has gained a great popularity since its first description and is currently considered to have at least the same efficacy compared to the traditional methods of performing liver biopsy from focal lesions [29]. EUS-LB has several advantages over the percutaneous and trans-jugular routes, including better safety profile [30], real-time high quality imaging of both liver lobes, and enabling access to the target focal lesions, thus avoiding intervening blood vessels and adjacent structures, as well as providing a real-time detailed view of the biopsy needle route during tissue acquisition through the liver [31,32,33]. Furthermore, EUS-LB is performed under sedation, guaranteeing reduced procedural anxiety and increased patient comfort [34,35]. Indeed, given the difficulty of achieving exact diagnosis of focal liver lesions by traditional imaging studies without histology in most liver lesions, specifically, in otherwise indeterminate hepatic solid masses, needle biopsy is mandatory to achieve final accurate diagnosis. To date, diagnostic utility of EUS-FNA/fine needle biopsy (FNB) in liver masses has been addressed by several studies. Overall, we could identify 13 papers in this field. Eleven papers have used FNA needles, while only two papers have used FNB needles. The first prospective study was performed by Nguyen et al. on 14 patients, who reported complete diagnostic yield with a zero complications rate [14], followed by a retrospective study conducted by Ten Berge et al., who examined the diagnostic yield of EUS-FNA for various liver lesions in 26 patients, which showed that an excellent overall diagnostic yield of 88.6% and a minor adverse event of transient low-grade fever occurred in one patient [36]. Notably, the largest study to date was conducted by DeWitt et al., who retrospectively reported the EUS-FNA yield in 77 patients, which showed an excellent diagnostic yield of 91%, without any reported complications [37]. A recent state-of-the-art review by Ichim et al. reported a high diagnostic accuracy of EUS-FNA of focal hepatic lesions, ranging from 80–100%, as this rate was non-inferior and even superior to biopsy under CT and US guidance [38]. Moreover, a retrospective analysis evaluating EUS-FNB in hepatic solid masses showed it to have a high diagnostic accuracy (89.7%), sensitivity (89.7%), specificity (100%), and sample adequacy (91.4%) for histology [39]. Finally, a very recently published prospective study by Ichim et al. addressing the diagnostic yield of endoscopic ultrasound-guided FNA in 48 patients with focal liver lesions detected by US, CT, or MRI found that in all but one patient with inadequate aspirate for appropriate histological analysis, the EUS-FNA was positive for malignancy in 47 patients (diagnostic yield of 98%). Most of the biopsies were taken from the left lobe (83%), while 17% were taken from the right lobe with similar technical success rate and without any reported immediate long-term complications. They concluded that EUS-FNA/FNB of focal liver lesions is highly accurate and safe and should not be limited only to cases of failure of percutaneous guided biopsies [4]. Recently, new fork-tip end-cutting needles have been evaluated in pancreatic solid lesions. Crinò et al. reported a similar diagnostic accuracy and safety profile compared to side-fenestrated needles; however, fork-tip needles provided a higher histological quality and lower needle passes number to achieve definite diagnosis [40]. A similar study demonstrated superiority of end-cutting 22G acquire needles as compared to 20G Procore FNB needles in pancreatic lesions [41]. Therefore, the newly introduced end-cutting needles might be more advantageous than the used biopsy needles. Further studies are needed to evaluate their safety and efficacy in solid liver lesions.

Although the data is scarce and still evolving, according to the current accumulating evidence, both needle types (FNA and FNB) are highly effective in achieving accurate diagnosis of focal liver lesions and have an excellent safety profile. Further studies are warranted to assess the diagnostic yield of FNA and FNB needles and their safety profile. Table 1 summarizes all studies reporting the utilization of EUS-FNA/FNB among patients with solid liver lesions.

## 5. EUS-Guided Solid Liver Lesions Treatment

EUS-guided solid liver lesions treatment has been recently gaining growing attention in the field of interventional gastroenterology and hepatology, as this new evolving treatment modality provides an effective acceptable alternative in difficult liver lesions, specifically lesions located in left and caudate lobes. The treatment options through EUS include ethanol injection, RFA, PDT, HIFU, laser thermal ablation, and brachytherapy [47]. Currently, the most common two treatment modalities are EUS-guided ethanol injection and RFA, which are mainly implicated in the treatment of primary and secondary liver malignant lesions. Table 2 reports all studies addressing the EUS-guided therapies in focal liver lesions. Overall, we could identify 10 publications, most of which deal with EUS-guided ethanol treatment in both HCC and liver metastasis. Overall, EUS-guided therapies for solid liver lesions were associated with an excellent technical success, very high therapeutic response, and minor adverse events.

### 5.1. EUS-Guided Ethanol Injection

The advantage of EUS-guided fine-needle ethanol injection is based on its ability to deliver a focused and targeted ethanol precisely to the tumorous liver lesion, thus avoiding damage to the adjacent non-tumorous liver parenchyma and minimizing treatment-related liver injury. Up to now, most studies reporting EUS-guided ethanol injection were performed among patients with HCC. Initial reports were on several case reports and described full technical success and complete resolution of HCC using 22G and 25G FNA needles, lacking procedure-related complications [48,49,50]. Nakaji et al. reported a very high-resolution rate for a follow-up period of 31 months and complete technical success in 12 patients with caudate lobe HCC and very mild procedure-related adverse events in 2 patients defined by transient low-grade fever [51]. While another study by Jiang et al. reported less promising results than the previous study, as only 30% (3 out of 10 patients with left lobe HCC) had complete HCC resolution for the follow-up period of 12 months, the technical success was still excellent, approaching 92% [52]. Nonetheless, the evidence currently available is still promising, despite the fact that it is only based on case reports and small case series and that one of the case series showed unexpectedly disappointing results. Therefore, more prospective studies with long-term follow-ups are warranted to precisely evaluate EUS-guided ethanol injection for HCC, since this therapeutic technique might be suitable for patients with HCC who are unfit for definitive treatment. Additionally, another implication for this treatment modality was reported in two cases of metastatic pancreatic adenocarcinoma to the liver. The first case achieved near complete resolution for a follow-up period of 1 month [53], while the second case had complete resolution after three treatment sessions, but the metastasis recurred after 24 months [54]. This method may constitute an alternative palliative therapeutic option for patients with liver metastasis who are not candidates for systemic chemotherapy. More studies are needed to evaluate safety and efficacy.

### 5.2. EUS-Guided Radiofrequency Ablation (RFA)

The application of EUS-guided RFA in patients with focal liver lesions is again reserved for HCC patients who are not candidates for definitive therapy and patients who failed previous minimally invasive therapies, such as trans-arterial chemoembolization (TACE) [57,58]. RFA is a low-risk minimally invasive procedure, which acts by delivering heat waves (in the range of 350–500 kHz) [59] that subsequently cause burning of the tumorous tissue, an effect that is mediated via coagulation necrosis [60]. A specifically designed needle tip electrode for performing EUS-RFA (EUSRA RF Electrode, STARmed, Koyang, Korea) with a designed internally cooled needle electrode was used for the first time in 2012 [61]. This EUSRA probe was present in 5- and 10-mm exposed tip electrode, as it connected to a radiofrequency generator, which provided radiofrequency power starting from 30 W and was accompanied by cooling to the internal needle, which subsequently lead to tumorous tissue ablation [61]. The efficacy and safety of EUSRA was first shown among patients with pancreatic neoplasms. Crinò et al. reported this technique in eight patients with pancreatic adenocarcinoma with complete technical success, high efficacy, and minor adverse events of mild-post procedural abdominal pain [62]. Similarly, another study by Song et al. reported a complete technical success with a high safety profile among six patients with unresectable pancreatic adenocarcinoma, who were treated by EUS-FNA using EUSRA electrode, delivering 20–50 W ablation power for 10 s [63]. To date, only one case report on the use of EUS-RFA used EUSRA in HCC. Armellini et al. reported a successful case of a 76-year-old female patient with cirrhosis, made complicated with HCC in the fourth liver segment, who underwent EUSRA with staring ablation power of 30 W for 30 min, without procedural related adverse events [64]. Accordingly, and given that RFA may represent a promising alternative therapeutic option in this setting, pilot human studies are needed to precisely assess the tumor response to EUS-RFA, as this modality might confer a promising therapeutic option for patients with unresectable HCC or metastatic liver cancer that is unamenable for systemic chemotherapy. Furthermore, a previous animal study assessing the coagulative effect of hybrid therapy of EUS-guided RFA and Cryotherpy combo-therapy in ex-vivo bovine liver (number 167) reached the conclusion that combining RFA and cryotherapy resulted in a better coagulative effect compared to either modality alone [65]. This study was subsequently confirmed by another animal study, showing satisfactory ablation areas without any complications [66]. Further studies are needed in humans to assess its efficacy and safety.

### 5.3. EUS-Guided Laser Ablation by Neodymium:yttium-Aluminum-Garnet (Nd-YAG)

This treatment modality was mediated via introducing laser waves through the EUS-needle directly to the tumorous tissue, leading to cell apoptosis and necrosis. Given the low power laser energy administered, this technique is considered minimally invasive and safe without causing normal adjacent liver tissue destruction [67]. To date, only two human studies have been reported. Di Matteo et al. reported the first case report of patients with caudate lobe HCC who failed treatment with TACE and who were treated next with EUS-guided laser ablation by Neodymium:yttium-aluminum-garnet with achievement of complete HCC resolution at 2 months follow-up without any reported procedure-related complications. Moreover, the technical success reported was 100% [55]. Recently, the largest study in this field was done by Jiang et al., who prospectively reported 10 patients with either HCC or colorectal carcinoma metastasizing to the caudate or left lobes of the liver, with complete resolution at 3 months without local recurrence with a high safety profile [56].

### 5.4. EUS-Guided Iodine-125 Brachytherapy

In the last several years, iodine-125 (125I) seed brachytherapy has gained popularity in the treatment of several cancers after showing that this treatment was associated with encouraging local control of solid tumors, such as head and neck cancers and prostate cancer [68,69,70,71]. The therapeutic effect of this treatment is achieved via the introduction of iodine-125 radioactive seeds to the tumorous liver tissue, leading to complete destruction. To date, only one study conducted by Jiang et al. addressed this treatment, which was performed on 26 patients with refractory malignant left-sided liver tumors. Thirteen of these patients were treated by EUS-guided iodine-125 seed implantation with high efficacy and safety, making EUS-guided iodine-125 brachytherapy an essential alternative, promising modality [52].

### 5.5. EUS-Guided High-Intensity Focused Ultrasound (HIFU)

HIFU is defined by thermal ablation. It has been used for surgical or trans-cutaneous approaches for various tumors [72]. However, the use of the EUS approach has only been reported in two animal models of liver tumor with complete necrosis of the lesions and without mention of adverse events [73,74]. Additional studies are needed.

### 5.6. EUS-Guided Photodynamic Therapy (PDT)

PDT is achieved through systemic infusion of photosensitizer material, which accumulates in tumorous tissue, followed by exposing patients to optic fiber, which produces light irradiation, resulting in reactive oxygen production, which activates the photosensitizer material, leading to tissue ablation [75,76]. PDT was reported earlier as treatment modality in solid tumors [77]. To date, PDT has been performed using the percutaneous approach. A previous retrospective study by Huggett et al. showed that PDT in pancreatic cancer was safe but was not associated with improved survival rate [78]. To date, only two animal studies on PDT administration under EUS guidance were performed by Chan et al., who assessed the efficacy and safety of EUS-guided PDT in porcine organs (pancreas, liver, kidneys, and spleen) and showed that PDT induced localized tissue necrosis in all organs but the pancreas was the most responsive organ to PDT, as the degree of necrosis was complete without significant adverse events [79]. Similarly, Yusuf et al. reported PDT as an effective option in inducing pancreatic tail necrosis in porcine models with high safety profiles [80]. However, to the best of our knowledge, no studies have been reported on EUS-guided PDT in human solid liver lesions; therefore, pilot studies are needed to elucidate its exact efficacy and safety in this setting.

## 6. Adverse Events

EUS-guided interventions for focal solid liver lesions diagnosis and management was shown to be associated with a minor adverse events rate, as was demonstrated consistently throughout the studies already mentioned. These adverse events mostly included self-limited puncture site bleeding, transient abdominal pain, and fever without the need for further therapeutic interventions. Among the 396 patients included in all studies involving EUS-guided liver biopsy for focal solid liver lesions, only four patients developed procedure-related adverse events, resulting in a complications rate of 1%. Among the 51 patients included in all studies that reported EUS-guided treatment for focal solid lesions, four patients experienced minor procedure-related adverse events, yielding a complications rate of 7.8%. Notably, no cases of procedure-related major adverse events or of mortality were reported in any studies, suggesting an excellent safety profile of this diagnostic and therapeutic intervention (Table 3).

## 7. Limitations

Our paper has several limitations. Firstly, it is restricted to articles published in the English literature and we might have missed data in this field published in other languages. Secondly, the evidence through the literature was based on small studies. Thirdly, it is unable to perform meta-analysis as most studies reported were case reports, case series, and retrospective studies.

## 8. Conclusions

Not far back, most interventions were in the hands of surgeons; however, as the imaging modalities have evolved, a significant volume of interventions have moved to the hands of invasive radiologist and, with the great developments in invasive endoscopy field, the gastroenterologists took command on a large volume of interventions that were outside the reach of endoscopy in the past. One of the biggest steps was probably in the EUS field, which has evolved from a merely diagnostic to a therapeutic modality, owing to its uniqueness of combining endoscopy with ultrasound in one hybrid scope. After the impressive advance of interventional EUS in pancreatic and biliary diseases, the liver was the next target, taking advantage of the high spatial resolution of EUS, proximity of the transducer to the organ of interest, minimal invasiveness, and excellent safety profile. Starting with EUS-RTE and progressing to real-time Doppler, CE-EUS, and CH-EUS images, the diagnostic yield of focal liver lesions was improved by assessing stiffness and vascular component of the lesion, as well as advancing towards targeted tissue sampling with better sample acquisition and very high diagnostic yield of live biopsy using FNA and FNB needles with excellent safety profiles. At a later stage, resources were directed towards a new still-evolving field of EUS-guided treatment of focal liver lesions, which were unsuitable for surgical intervention or lesions that were unresponsive to treatment, such as TACE, particularly for lesions in the left and caudate lobes. Several EUS-guided therapies have been already reported in humans and some were tried in animal models. All were shown to provide an effective, safe, acceptable alternative in treating focal liver lesions (Table 3). Possibly, in the close future, some of our first line approaches will change in favor of the above, reporting rapidly growing and expanding applications to cover almost all the aspects of diagnostic and therapeutic hepatology.

## Figures and Tables

**Table 1:** Studies reporting endoscopic ultrasound (EUS)-guided liver fine needle aspiration/ fine needle biopsy (FNA/FNB) in focal liver lesions.

	Type of Study	Patients No.	Tissue Acquisition	Diagnostic Yield, *n* (%)	Complications, *n* (%)
Nguyen et al. [14]	Prospective	14	FNA	14 (100)	0
TenBerge et al. [36]	Retrospective	26	FNA	23 (88.6)	1 (3.8) *
DeWitt et al. [37]	Retrospective	77	FNA	79 (91)	0
Hollerbach et al. [42]	Prospective	33	FNA	31 (94)	2 (6.1) **
McGrath et al. [43]	Prospective	7	FNA	7 (100)	0
Sing et al. [12]	Prospective	9	FNA	8 (88.9)	0
Sing et al. [13]	Prospective	26	FNA	25 (96)	0
Crowe et al. [44]	Retrospective	16	FNA	12 (75)	0
Prachayakul et al. [45]	Retrospective	14	FNA	14 (100)	0
Oh D. et al. [33]	Prospective	47	FNA	42 (90.5)	0
Ichim et al. [4]	Prospective	48	FNA	47 (98)	0
Lee et al. [46]	Prospective	21	FNB	19 (90.5)	0
Chon et al. [39]	Retrospective	58	FNB	52 (89.7)	1 (1.7) ***

* Low-grade transient fever. ** Self-limited bleeding. *** Bleeding complication that was controlled with endoscopic hemostasis.

**Table 2:** EUS-guided available therapies for focal solid liver masses.

EUS-Guided:	Study Type	Patients No.	Technical Success (%)	Lesion Location	Therapeutic Response	Complications, *n* (%)
**Ethanol therapy in hepatocellular carcinoma**						
Nakaji et al. [48]	Case report	1	100	Segment 8	Complete	0
Lisotti et al. [49]	Case report	1	100	Segment 2	Complete	0
Nakaji et al. [50]	Case report	1	100	Segment 3	Complete	0
Nakaji et al. [51]	Retrospective	12	100	Caudate lobe	Complete	2 (16.7) *
Jiang et al. [52]	RCT	10	92	Left lobe	Partial (30%)	0
**Ethanol therapy in liver metastasis**						
Hu et al. [53]	Case report	1	00	Left lobe	Near-complete	1 (100) *
Barclay et al. [54]	Case report	1	100	Left lobe	Complete	1 (100) **
**Ablation by Nd-YAG**						
Di Matteo et al. [55]	Case report	1	100	Caudate lobe	Complete	0
Jiang et al. [56]	Prospective	10	100	Left lobe	Complete	0
**Iodine-125 brachytherapy**						
Jiang et al. [52]	RCT	13	92	Left lobe	Near-complete	0

* Transient low-grade fever. ** Self-limited subcapsular hematoma.

**Table 3:** Summary of efficacy and safety of EUS-guided intervention in solid liver lesions.

Procedure	Efficacy	Complications	Mortality
Tissue diagnosis (EUS-LB)	High	Mild to moderate	None
EUS-guided solid liver lesions treatment			
Ethanol therapy Laser ablation by Nd-YAG Iodine-125 brachytherapy	High High High	Mild Mild Mild	None None None

Moderate complications: Bleeding needed endoscopic homeostasis; mild complications: minimal self-limited bleeding, transient fever, and mild pain.

## Abbreviations

EUS	Endoscopic ultrasound
CT	computerized tomography
MRI	magnetic resonance imaging;
CE	contrast-enhanced
US	ultrasound
FNA	fine needle aspiration
RTE	real-time elastography
RFA	radiofrequency ablation
HIFU	high-intensity focused ultrasound
PDT	photodynamic therapy
HCC	hepatocellular carcinoma
UCA	ultrasound contrast agents
CH-EUS	contrast harmonic-EUS
EUS-LB	EUS-guided liver biopsy
FNB	fine needle biopsy
TACE	trans-arterial chemoembolization
